# Emergence of epidemic diseases: zoonoses and other origins

**DOI:** 10.12703/r/11-2

**Published:** 2022-01-18

**Authors:** Robin A Weiss, Neeraja Sankaran

**Affiliations:** 1 Division of Infection & Immunity, University College London, London, UK; 2 The Descartes Centre for the History and Philosophy of the Sciences and the Humanities, Utrecht University, Utrecht, The Netherlands

**Keywords:** Infectious diseases, origin, emergence, zoonosis, history of disease

## Abstract

Infectious diseases emerge via many routes and may need to overcome stepwise bottlenecks to burgeon into epidemics and pandemics. About 60% of human infections have animal origins, whereas 40% either co-evolved with humans or emerged from non-zoonotic environmental sources. Although the dynamic interaction between wildlife, domestic animals, and humans is important for the surveillance of zoonotic potential, exotic origins tend to be overemphasized since many zoonoses come from anthropophilic wild species (for example, rats and bats). We examine the equivocal evidence of whether the appearance of novel infections is accelerating and relate technological developments to the risk of novel disease outbreaks. Then we briefly compare selected epidemics, ancient and modern, from the Plague of Athens to COVID-19.

## Introduction

*Healthy populations are all alike; each epidemic emerges in its own unhappy way.*

To parody the opening sentence of Tolstoy’s *Anna Karenina* serves our main message, namely that the origins of novel epidemic diseases are diverse^1,2^. Whereas popular commentaries on pandemic risk tend to attribute nearly all newly emerging human infections to cross-over from animals^3^, we stress that, though certainly significant, animals are far from being the only source of pandemics, either today or in history. Indeed, as microbiologist Rita Colwell observed, more often than not “human activities drive emergence of disease”^4^. In this essay, we concur with her view and show how the odds of an infectious disease outbreak becoming epidemic or pandemic depend on various microbiological, environmental, and social factors that interact in complex and often unpredictable ways^5–7^.

Although about 60% of novel epidemic diseases in recent decades are indeed zoonotic in origin, the remaining 40% have other origins^2^. To cite three familiar non-zoonotic infections, chickenpox virus has coexisted with its pre-human and human hosts throughout the course of primate evolution^8^, whereas two of the most prevalent pandemic diseases of the nineteenth century—cholera^5,9^ and tuberculosis^10^—originally came to humans from microbes living in water and soil, respectively.

Two examples of outbreaks that appeared almost simultaneously in 1976 illustrate how previously unknown pathogens may suddenly emerge in quite different environments. Whereas the Ebola breakout in the small village of Yambuku in the Democratic Republic of Congo probably came from bats via an intermediate primate host^11^, the episode of Legionnaires’ disease arose from bacteria (*Legionella pneumophila*) in an air-conditioning plant in downtown Philadelphia—no animals except humans were involved^12^. Interestingly, Ebola is seen as a huge threat to humankind whereas Legionnaires’ disease rarely hits the headlines anymore. Since 1976, however, there have been about 34,000 human Ebola cases recorded and over 50,000 cases of Legionnaires’ disease^13^.

### Taking stock of infectious diseases today

Fifty years ago, the proportion of deaths due to infection had greatly diminished, and life expectancy had increased because of better nutrition, improved hygiene, childhood immunizations, and antibiotics. In 1980, the World Health Organization (WHO) announced the eradication of smallpox, and there was great optimism that humans were well on the way to conquering infectious disease. But new threats soon appeared and dashed such hopes. AIDS took off pandemically in 1981 and microbial resistance to antibiotics began to be recognized. Old threats re-emerged: malaria when mosquitos became resistant to insecticides, and the severity of tuberculosis grew hand-in-glove with the rise of AIDS. Since then, the WHO has had to tackle novel outbreaks of highly pathogenic strains of avian influenza A virus, severe acute respiratory syndrome (SARS), Ebola, Zika, and COVID-19.

**Figure 1.**
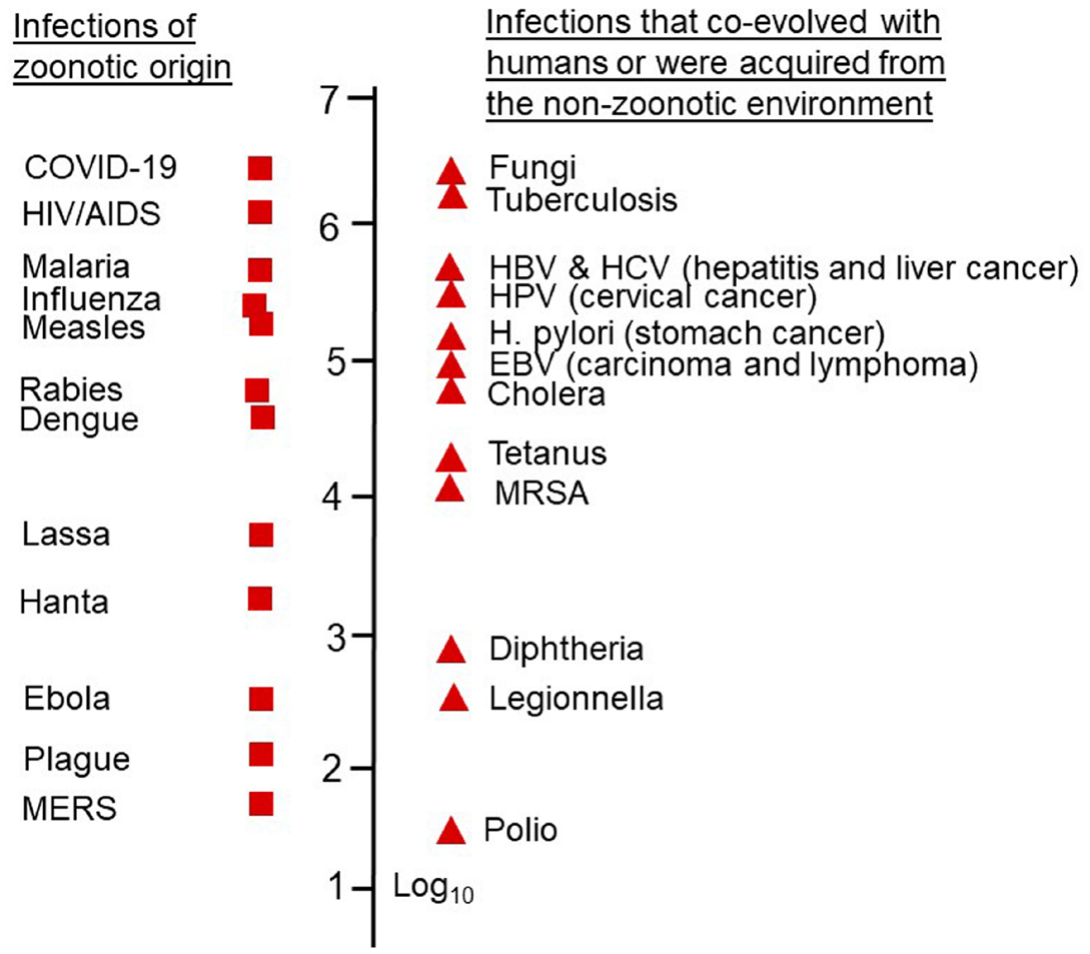
A “Richter” scale of estimated global mortality in 2020. Endemic diseases, including tuberculosis, cancer-causing viruses, bacteria, and malaria, account for a high proportion of mortality due to infections. But COVID-19 and fungal infections (including the recent *Candida auris* pandemic) top the list for the year 2020. Data are based on estimates from the World Health Organization and Our World in Data. EBV, Epstein–Barr virus; HBV, hepatitis B virus; HCV, hepatitis C virus; HPV, human papillomavirus; MERS, Middle East respiratory syndrome; MRSA, Methicillin-resistant *Staphylococcus aureus*.

Figure 1 shows approximate estimates of global mortality due to selected infectious diseases in 2020. Although COVID-19 was unknown until December 2019, it became a leading contributor to infection mortality in 2020, but what tends to be overlooked is that a similar number of deaths was caused by fungal infections, which includes the recently emerged *Candida auris* pandemic^14^.

Mortality is not necessarily the best measure of the impact of an infectious disease. Even when they don’t kill, diseases can greatly affect quality of life, not only of an affected person but of society more broadly. Indeed, relatively small outbreaks in history have led to massive social, economic, and cultural changes (see, for instance, examples in Snowden^15^). Another measure of disease impact is disability-adjusted life years (DALYs), which represents the years of potential life lost due to premature deaths and years of productive life lost due to disability. On the other hand, a death due to, say, malaria or rotavirus in infancy, though a tragedy for the family concerned, has less societal and economic impact than a young adult who dies from AIDS or in a traffic accident.

In this article, a virologist (RAW) and a historian of microbiology and medicine (NS) examine the emergence of infectious diseases mainly from the “germ’s eye view”. We review data about origins of infections on the basis of molecular and genomic sources and relate them to what is known from historical sources. We discuss some general epidemic concepts followed by a consideration of virulence and susceptibility to infection and disease. We then consider zoonoses and other origins and how changes in human ecology and technological development have altered epidemic risks. We end by briefly reviewing historical examples of epidemics and pandemics in the context of more recent ones.

## Epidemic concepts and methodology of transmissible diseases

A few words on terminology may be useful at this point. We refer to disease-inducing microbes as *pathogens* and to the intensity of the disease they cause as their *virulence*. An *endemic* infection is one that is chronically present within a given population, maintained by transmission to individuals not previously exposed to it (for example, infants). An *epidemic* infection represents an outbreak in which the number of infected people suddenly increases and becomes a *pandemic* when it spreads to several continents^16^. In 2010, the WHO introduced as an additional criterion in defining a pandemic, the condition that it had to be a “new” infection. However, this has only added confusion to the issue, for it does not sufficiently define what this newness entails.

*Zoonosis* (pl. zoonoses) is the phenomenon by which an infectious agent transfers from a natural animal reservoir or an intermediate animal host to humans^17^. Once the agent acquires the capacity for temporary or permanent human-to-human infection, it is better referred to as an infection of zoonotic origin. Single events may trigger a cross-species outbreak of zoonotic origin—or of presumed zoonotic origin if the source species has not been identified; for instance, anecdotal evidence in the Ebola outbreak in 1976 suggests that a man brought home a sick monkey to Yambuku village as bushmeat^11^.

A relatively new term, *syndemic*, initially referred to the simultaneous and related occurrence of two infectious diseases that act synergistically to magnify the burden of disease. The re-emergence of tuberculosis in conjunction with AIDS is an example of a particularly serious syndemic problem^18^. The syndemic concept has since been expanded to include the interactions of a pathogen with non-communicable co-morbidities—such as mental health and poverty—both in individuals and in societies^19^; this is especially pertinent to COVID-19^20^. Thus, differing social and medical conditions in human populations may determine their vulnerability to novel infections.

**Figure 2.**
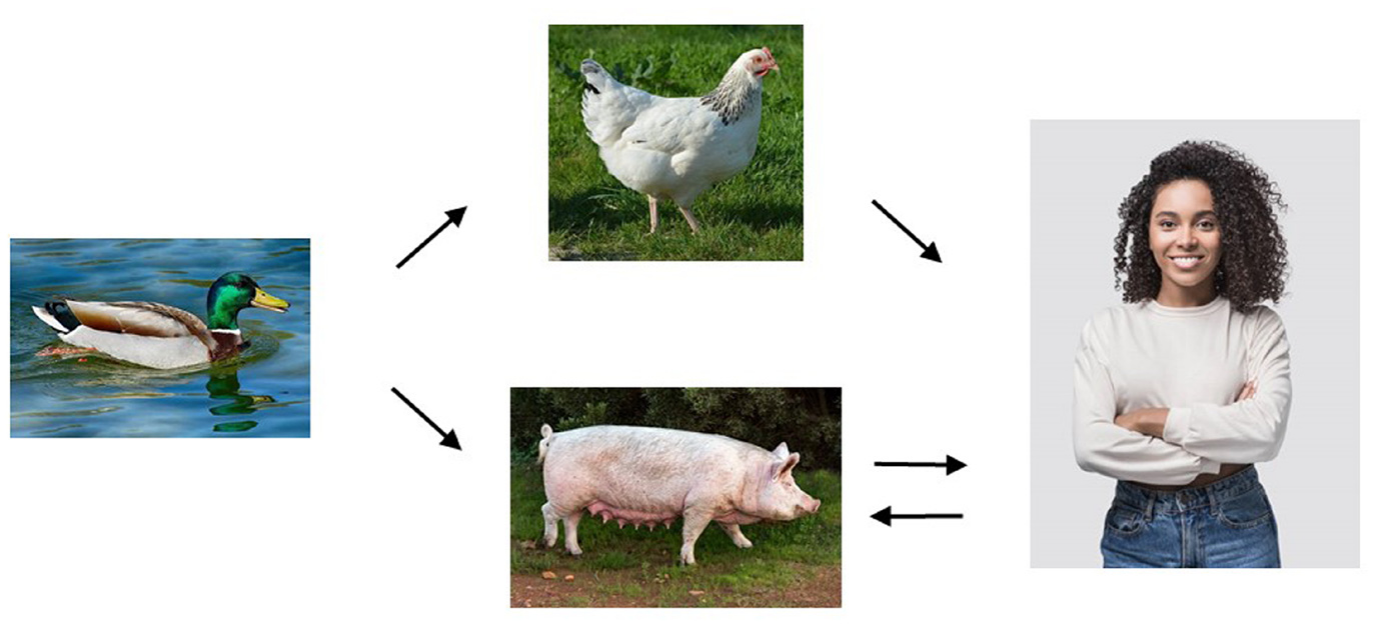
Cross-species transmission of influenza A viruses (IAVs). The typical reservoirs of IAVs are wild waterfowl where the virus replicates as an intestinal infection transmitted as a water-borne infection via the cloaca. Upon transfer to mammals and chickens, the virus adapts to subtly different sialic acid receptors on the cell surface which promote respiratory infection and aerosol transmission. IAVs may undergo genetic reassortment with endemic strains already present in an intermediate host to produce new variants more likely to transfer to humans. In turn, humans can be a reservoir for reintroduction to animals, as seen in the influenza pandemic in 2009 when pig farmers were a source of outbreaks in their livestock. (Copyright-free images from Pixabay.)

The most frequent usage of the term *spillover* denotes an infection that has moved from an animal source to cause an outbreak in humans, which may then take on epidemic proportions because of human-to-human transfer^3^. Of course, diseases can and do spill over from humans to other animal species; spillover in both directions is depicted for influenza A virus in Figure 2. Similarly, after SARS-CoV-2 reached Europe, it spilled over from humans to mink^21^. Spillover from humans to animals is often called reverse zoonosis or zoo-anthroponosis.

A second, lesser use of *spillover* refers to the explosive growth of an animal population around human habitation, so that a high virus load spills over in excreta, food, or house dust, setting off a human epidemic even though direct spread from human to human is less frequent. Two examples are the population bursts of the commensal multimammate rat *Mastomys natalensis* in West Africa promoting the dispersal of the arenavirus, causing Lassa fever^22^, and the release of hantaviruses due to seasonally abundant mice and voles, causing acute pulmonary and renal syndromes^23^.

A third sense of *spillover* (actually its earliest usage) denotes the spread of an infection from one tissue to another within the infected host. Examples include poliomyelitis, when the spillover of poliovirus from an asymptomatic infection in the gut to the central nervous system causes paralysis, and shingles, when varicella-zoster virus becomes activated from its latent form in sensory neurons and spills into the skin.

Pathogens transfer from one individual to another by varied routes: respiratory, fecal–oral, skin contact, parenteral, and sexual and via arthropod vectors. The same pathogen may adopt different modes at different stages of an epidemic. For example, plague outbreaks caused by *Yersinia pestis* are augmented by both insect-borne and respiratory infections. A new outbreak is usually zoonotic, sparked by rodent fleas biting humans, but is then sustained by spread from one human to another either via human fleas and lice—as happens with bubonic plague—or by the respiratory route, causing pneumonic plague.

Non-persistent infections rely on a pool of susceptible individuals to keep going in the host population. Survivors are usually protected from reinfection by immune memory, at least temporarily and often for the rest of their lives. Immunization achieves similar protection without imposing the toll taken by the disease. A pandemic will not occur when a high proportion of people acquire immunity—called *herd immunity*—although smaller-scale epidemics may break out among the susceptible individuals^24^. Thus, before vaccines were developed for common infections such as measles, diphtheria, and pertussis, regular epidemics occurred among children^25^.

When an infection reaches a naïve community (that is, a population with no prior exposure or immunity among adults), the result can be a demographic catastrophe. The 1521 co-introduction of smallpox and measles to the New World, where the population of indigenous peoples fell by 90% over the ensuing 100 years, is one historical example^26^. A sinister consequence of this population implosion was the development of the slave trade to provide labor for the plantations.

The power of genome sequencing and phylogenetic analysis has greatly added to knowledge from historical, ecological, and epidemiological studies regarding the timing and origins of human infections^27,28^. Even when ancient remains of pathogens are not available, molecular clock estimates of the timing of divergence from a common ancestor can illuminate origins^29^; for instance, rinderpest of cattle and measles in humans parted company around 2,500 years ago^30^ even if human infections occurred previous to this time. Molecular clock simulations are also useful for surveillance of the emergence of recent pandemics, such as COVID-19 and the “near misses” of related viruses that have not colonized humans^31^.

### Virulence and determinants of host susceptibility

Virulence does not always benefit the onward transmission of the infectious agent. Compare two recently emerged influenza strains: The 2005 epidemic of H5N1 avian influenza was highly pathogenic in both chickens and humans but did not spread between humans. By contrast, during the 2009 H1N1 swine-origin influenza pandemic, some 600 million people became infected, but most had mild symptoms and “only” 284,000 died^32^. A disproportionately high number of those who died, however, carried a rare genetic variant of the IFTM3 host defense gene, which made them susceptible to severe disease^33^. Clohisey and Baillie^34^ quipped that, for influenza, “patients with life-threatening disease are, by definition, abnormal hosts”.

The virulence of an infection after transfer to a new host species is unpredictable. It may increase enormously, as in the case of the myxomatosis poxvirus in European rabbits, compared with its original host, the South American cotton-tail rabbit^35^. On the other hand, cowpox is less virulent in humans than in cattle and possibly in its reservoir species, bank voles (*Myodes glareolus*)^36^.

## Zoonoses: from spillover to new normal

As an invasive species with a global population of 7.8 billion, we humans may ourselves be regarded as a plague upon planet Earth, driving climate change and affecting the environment globally^37^. The most important factor for the emergence of human infections of pandemic proportions has been the dramatic increase in the size and density of the human population ever since the development of agriculture. Humans host a greater number of infectious diseases than our great ape relatives, having acquired them during different phases of our “progress” toward civilization^5^, from migration out of Africa, increased contact with various animal species because of hunting, herding, and domestication, and exposure to animals that adopted human habitats. It is worth noting, however, that we have little awareness of the hundreds of different types of viruses, microbes, and parasites to which we are exposed from the animals around us because few of them are serious pathogens and even fewer adapt to human-to-human transmission^38^.

### Direct zoonoses

Rabies exemplifies the classic zoonotic infection. The vast majority of human cases are acquired from dogs, in which rabies became endemic many centuries ago, presumably having crossed over from a closely related lyssavirus of bats. More than 99% of human infections are fatal unless treated immediately after exposure. Although safe and efficacious vaccines that protect dogs and humans exist and canine immunization is reducing human incidence, at least 50,000 humans are estimated to die from rabies annually exacting an economic toll of $8.6 billion (USD)^39^. The burden of disease is disproportionally borne by children in poor, rural populations of Africa and South Asia. Given that the annual death toll from rabies far exceeds that of Ebola virus, why don’t we place as much effort on controlling rabies as Ebola? One reason is that rabies is no longer perceived as a pandemic threat since humans are a “dead-end” host without onward transmission.

### The new normal: stepwise establishment of epidemics of zoonotic origin

Predicting which pathogen has pandemic potential is tremendously important, but despite all the advances in modeling, prediction remains inexact. Many factors affect the steps toward the establishment of an infection of zoonotic origin^40^. Even when spillover occurs, most episodes are restricted to local outbreaks or to transcontinental epidemics such as SARS that soon fizzle out. It is on those rare occasions that the pathogen adapts to human-to-human transmission that the spillover becomes long-term or permanent; it becomes the “new normal”, as in the case of novel influenza strains and SARS-CoV-2. When the infected host can pass the infection on before symptoms appear or when a high proportion of infected individuals do not show symptoms, the agent can spread by “stealth”^7^. Thus, although much emphasis in pandemic prediction is placed on surveillance of initial exposure to novel pathogens, subsequent steps in a novel pathogen’s adaptation to humans play an equal if not more important a role for an outbreak to become pandemic (Table 1).

**Table 1.** Steps in the establishment of a novel infection from its introduction to becoming endemic in the human population.

Event	Potential bottlenecks or methods of control
Exposure to agent	Proximity to animal reservoir or environmental source
Infection by agent	Only a minority infect humans, who may be a dead end
Human-to-human transmission	Onward transmission subject to host restriction factors
Epidemic spread	Reproductive rate R_0_ > 1. If R_0_ < 1, epidemic becomes self-limiting.
Pandemic spread	International dispersal blocked by quarantine measures
Endemic status	Prevalence in population modulated by herd immunity
Control and eradication	Vaccines and anti-microbial drugs
Emergence of variants that increase transmission, escape immune control, and affect virulence	

Many of the well-known childhood infectious diseases had a zoonotic origin in antiquity. The two best-understood examples are the RNA morbillivirus causing measles and the DNA variola virus causing smallpox. The closest animal relative to measles virus was rinderpest of cattle (now eradicated through a combination of immunization and the management of animal populations), whereas variola virus is closely related to camelpox, which itself probably transferred from a rodent poxvirus. The *variola major* strain of smallpox virus probably diverged from camelpox around 1,700 years ago^41^. Domestication of cattle and camels occurred several thousand years earlier, providing increased opportunities for zoonotic transfer events, but the maintenance of these viruses in humans without continuing dependence on the animal reservoir awaited the growth of large and dense human populations.

Nowadays, successful zoonotic infections such as novel influenza strains (Figure 2) tend to become pandemic soon after their introduction. SARS-CoV-2 became pandemic within 3 to 4 months of the estimated time of the animal-to-human infection event^31^. Such rapidly adapting viruses tend to have a broad host range capable of infecting many species. Not all pandemics of zoonotic origin take off so rapidly; it took HIV-1 group M about 50 years to emerge pandemically, from the likely initial chimpanzee-to-human transfer event^42^.

### Is the frequency of zoonotic epidemics increasing?

**Figure 3.**
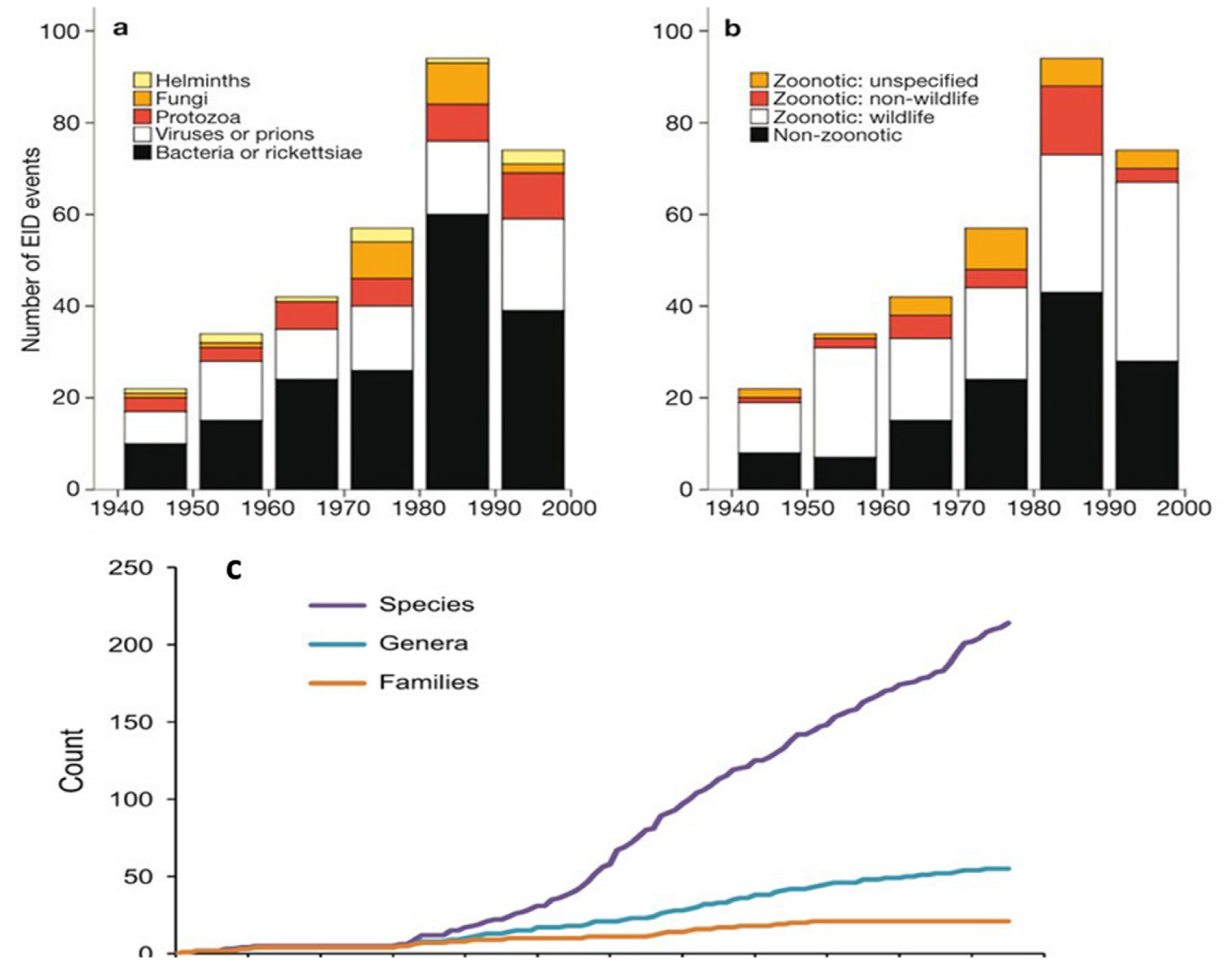
Proportions of novel infectious diseases in humans from 1940 to 2000. Classified by (**a**) type of microbe and (**b**) origin. (**c**) Cumulative number of novel zoonoses by RNA viruses between 1900 and 2016. Figure (**a**) and (**b**) was reprinted with permission from Springer Nature, Global trends in emerging infectious diseases, Jones *et al*. 2008^2^. Figure (**c**) was reproduced with permission from Mark Woolhouse^38^. EID, emerging infectious disease.

It is commonly stated by environmental campaigners that our wanton destruction of the non-agricultural environment greatly increases the risk of novel epidemic zoonoses but this assumption is open to challenge. Indeed, the same data have been used to make contradictory claims: The opening sentence in Dobson and colleagues^43^, for instance, states that “For a century, two new viruses per year have spilled over from their natural hosts”. In contrast, Rohr and colleagues^44^ claim that “Infectious diseases are emerging at an unprecedented rate”. Both articles cite as evidence the same 2008 landmark paper by Kate Jones and colleagues^2^. Figures 3a and b (reproduced here from that paper) do not unequivocally show an increasing rate of novel infections except for bacterial and rickettsial infections in the 1980s and a modest increase in zoonoses from wildlife.

Another study of large data sets^45^ detected an increase in overall outbreak events of infectious diseases, including zoonoses, between 2008 and 2013 but a decrease in the total number of affected people. More recent analyses by Mark Woolhouse and colleagues^46,47^ focused specifically on novel zoonotic RNA viruses have found that since 1950 the rate of detection has been broadly constant (Figure 3c). Although there might be a slight increase in the frequency of outbreaks since the turn of the millennium, this may be due to greater ascertainment as surveillance has improved and intensified. Thus, while novel zoonotic infections continue to occur, the rate of emergence remains controversial; it might be speeding up but not dramatically.

## Ecology of emergence

### Maintenance of infections in prehistoric societies

When humans lived in small groups as gatherer-hunters, they were subject to infectious diseases, although there was little opportunity for pandemic emergence. Endemic pathogens that co-evolved with humans, such as the herpesviruses, Epstein–Barr virus, and chickenpox (varicella-zoster) virus, are well adapted to vertical transmission across host generations by establishing lifelong reservoirs within the human body^5^. Thus, chickenpox virus establishes a latent reservoir in sensory neurons but can re-emerge as zoster (shingles) decades later, spilling over into the skin. In this way, contact with a grandmother with shingles can trigger a chickenpox epidemic spread by skin contact or by the respiratory route among children two generations younger. Zoonotic infections crossed over to humans in the Paleolithic and earlier eras for instance, malaria from apes^48^. As the infections became endemic, they diverged from their non-human progenitors. We see the same phenomenon in modern times, whether the adaptation to humans has been slow as with HIV or rapid as with influenza and SARS-CoV-2. Infections that do not persist in recovered individuals will only persist in the human population, either if there is frequent spillover from a non-human source or if the population is sufficiently large and dense to afford continual access to new naïve individuals^25^.

One important bacterial pathogen that was introduced to humans in prehistoric times is the agent of tuberculosis, *Mycobacterium tuberculosis*. This organism belongs to a group of obligate parasitic bacteria, the *Mycobacterium tuberculosis* complex (MTBC), which are related to certain mycobacteria found in soil, where they are associated with amoebae, and which were probably their original source^49^. MTBC DNA has been isolated from human skeletons in the Old and New World dating back at least 9,000 years. It may have come to humans during the Paleolithic period as far back as 70,000 years ago^10,50^, although a new metagenomic analysis indicates a more recent Neolithic common ancestor to modern strains^51^. MTBC bacteria established persistent infections well suited to survival in small host populations with a low transmission rate and became pandemic only in recent centuries because of crowded and poorly ventilated living conditions. Phylogenetic trees of MTBC indicate that *M. bovis* and other animal strains are secondary derivatives of human *M. tuberculosis*^52^.

### Globalization of infections

As humans spread out of Africa to all habitable continents, they encountered new environments and animal species affording opportunities for diverse microbes to adopt the human host. In the historic era, human globalization brought specific pathogens to new regions giving rise to pandemics such as the Black Death, smallpox, and syphilis (discussed below). Diamond^53^ noted that these diseases mainly spread westwards along latitudes rather than longitudes (for example, along the Silk Road from East Asia to Europe and across the Atlantic Ocean).

In addition to human mobility, the migration of reservoir species plays a role in the dispersal of infectious disease. Avian influenza virus strains maintain reservoirs in aquatic, migratory wildfowl whence it spreads to domestic and semi-domesticated fowl and mammalian livestock. Bats may also fly great distances and temporarily camp in different roosts, permitting exchange of pathogens^54^. Arthropod vectors of infectious diseases have expanded their geographic range because of both increased human movement and global climate change. The anthropophilic species of mosquito, *Aedes aegypti* and *A. albopictus*, which breed by preference in urban environments, have become the chief purveyors of viruses such as yellow fever, dengue, chikungunya, and Zika^55^, although these viruses originated centuries ago as sylvatic infections in forests.

### Deforestation and afforestation

Another popular notion among environmental groups concerned about infectious diseases—for example, the Center for Biological Diversity and the EcoHealth Alliance—is that today’s novel infectious diseases originate from exotic species that are gaining access to humans chiefly through our invasion and destruction of pristine forest habitats. Closer examination, however, indicates that new outbreaks more commonly involve wild animals that live in proximity to humans^56^ or originate as bacterial zoonoses from livestock^57^. The interface between wild animals, domesticated livestock, and humans is complex^58^. Although changes in land use and natural habitat erosion clearly play an important role in zoonotic risk^59^, animal species remote from human habitats are not the most frequent sources.

Let us consider bats (*Chiroptera*)^60^. Contrary to populist accounts, most bat species are not recent migrants to human habitats as a result of deforestation; many have long been anthropophilic, often making use of human constructs for roosts. For instance, the main field site for studies of SARS-related coronaviruses in horseshoe bats by the Wuhan Virology Institute is a disused mine shaft^61^. As Rulli and colleagues^62^ show, the more developed and populous landscapes in China, Southeast Asia, and Europe favor horseshoe bats that are reservoirs for SARS-related coronaviruses.

**Figure 4.**
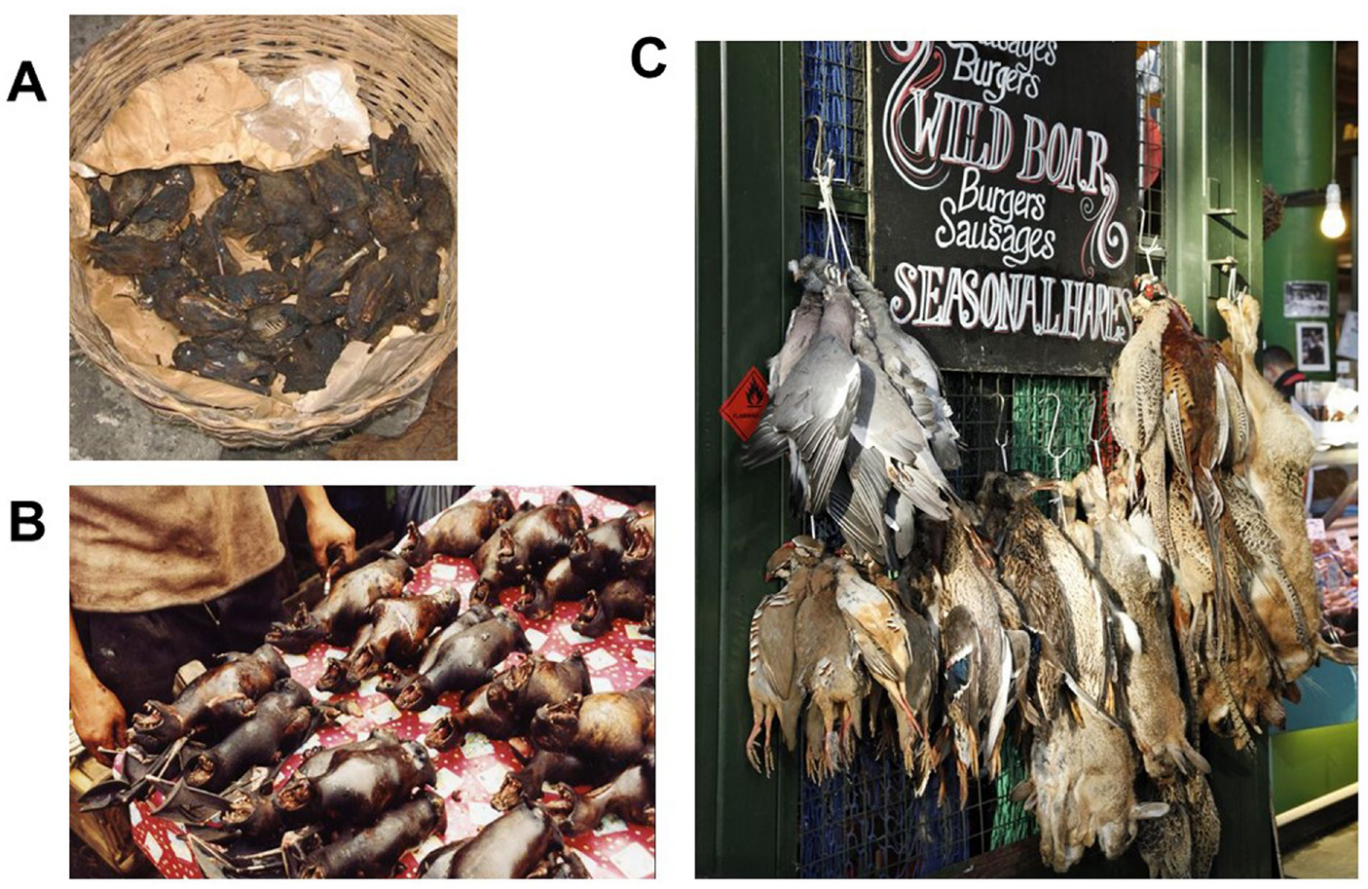
The “bushmeat trade” on three continents. (**A**) *Eidolon helvum* fruit bats for sale at a market in Accra, Ghana. (**B**) Cooked *Pteropus* sp. fruit bats on a market stall in Sulawesi, Indonesia. (**C**) Game for sale at Borough Market, London, UK. The potential zoonoses from the fruit bats include filoviruses, lyssaviruses, and paramyxoviruses as discussed in the text; the potential zoonoses from unscreened game species in Borough Market include influenza A virus (mallard duck), *Psittacosis* (pigeon), *Salmonella* (partridge), *Leptospirosis* (rabbit and hare), and hepatitis E virus (wild boar). Photographs courtesy of David T.S. Hayman (**A**, **B**) and Robin A. Weiss (**C**).

Fruit bats (flying foxes) across Africa, Asia, and Australia are also naturally attracted to cultivated land and sometimes are trapped and sold for food (Figure 4). The African straw-colored fruit bat, *Eidolon helvum*, is a host to coronaviruses, lyssaviruses (related to rabies), filoviruses (Ebola and Marburg), and paramyxoviruses of the henipavirus group related to Nipah virus. It is frequently found in urban roosts (for example, Accra and Ibadan) but is seasonally migratory between native forest and city trees^63^. Small outbreaks of Nipah virus infection frequently occur from *Pteropus* feeding on date palm sap and farmed fruit in Bangladesh^64^ and Kerala, India^65^, which are two of the most densely populated agricultural areas worldwide. There are 17 stable roosts of mixed *Pteropus* species in Sydney, although individual bats migrate long distances and visit other roosts in the bush^54^, permitting exchange of potential pathogens. Thus, although it behooves us to scan the far horizon for pandemic preparedness using a panoply of modern approaches^66^, the next “exotic” epidemic might well arise in an orchard or the belfry of a church or temple.

It has been argued that bats under environmental stress may have more active viruses available for cross-species transmission^60^ and that their high metabolic state may aid this process^67^. Moreover, bats tolerate viral infections well^68^. Mollentze and Streicker^69^, however, reckon that rates of zoonosis vary little between host taxonomic orders when the number of different species is taken into account. In that case, *Rodentia* and *Chiroptera* are at the forefront of zoonotic events because these orders comprise the most species.

The welcome rewilding of forests and their fauna, albeit without large carnivores that balance population growth of prey, has led to more frequent infection by well-known zoonoses. Lyme disease caused by *Borrelia burgdorferi* is transmitted via *Ixodes* ticks of deer in the Northeastern US^70^, and wild boar in Europe frequently carry hepatitis E virus^71^. Westerners patronizingly call meat from wild animals “bushmeat” when captured in Asia or Africa and frown upon its consumption yet seem unperturbed by the sale of “game” in our own cities (Figure 4).

The majority of emerging or re-emerging zoonoses come from domestic animals and commensal wildlife; these include almost all recent zoonoses of bacteria, fungi, and DNA viruses. That leaves the RNA viruses, which lie behind most of the “newsworthy” outbreaks and epidemics. Since afforestation near human habitats increases well-known zoonoses, deforestation of tropical forests may increase the arrival of novel, sylvatic zoonotic agents into the human ecosystem. We should therefore examine whether the twenty-first-century viruses at high risk of spawning epidemics of pandemic potential are accurately attributed to deforestation.

Recent outbreaks and epidemics of RNA viruses include influenza A viruses (H5N1 and H1N1), paramyxoviruses (Nipah and Hendra), coronaviruses (SARS, Middle East respiratory syndrome [MERS], and COVID-19), flaviviruses (West Nile and Zika), and filoviruses (Ebola and Marburg), most of which are discussed in more detail later. Only the filovirus outbreaks appear to be directly related to sylvatic sources remote from developed land. That the Ebola and Marburg viruses have not become pandemic is partly because of their short incubation period and because direct contact is required for transmission. The Ebola epidemic of 2013–2014, however, did spread to cities; its origin was traced to bats roosting in a village in the Guéckédou province in Guinea^1,72^. Curiously, further minor outbreaks have also occurred in Guéckédou: Ebola in June 2021 and Marburg in July–August 2021^73^. The 2018–2020 Ebola epidemic in eastern Democratic Republic of Congo may well have come from forest bats because of the upheaval and human conflict in that region^74^. Overall, despite real concerns of the erosion of pristine forests, there is no compelling evidence that deforestation or human invasion of forests is the major driver for the emergence of RNA viruses.

### The impact of modern technological change

From Paleolithic hunting with spears to animal domestication and from early agriculture to modern logging methods, technological development has changed human exposure both to animal infections and to environmental microbes. There is an extensive literature in the history of medicine and technology on this topic^75,76^. Here, we cite just four examples in which advances in technology have played a role in outbreaks of infectious disease:

A) The invention of hypodermic injection equipment in the early twentieth century, which came as a boon for delivering drugs and vaccines, proved to be a public health disaster when syringes and needles were reused without adequate sterilization. It galvanized the spread of hepatitis C virus and other parenterally transmitted infections^77^. Injections were also a route of HIV-1 dispersal in Africa in pre-pandemic days^42^, similar to its spread among intravenous drug users in the West much later.

B) The introduction of mechanical extraction of neural tissue from bovine carcasses and its recycling in cattle feed represented a type of cannibalism forced on herbivores by humans. It led to “mad cow disease”, a fatal spongiform encephalopathy caused by altered prion proteins^78^. The UK government held that consumption of beef products would not transfer infectious prions to humans since scrapie, a similar disease that has been widely endemic in sheep for at least a century, had never done so. Again, the odds for zoonosis lay in the molecular detail: the bovine prion has a methionine residue at position 129 shared with human prions but not with ovine prions. In 1996, spillover to the human population in the UK was detected as variant Creutzfeldt–Jakob disease^79^.

C) Legionnaires’ disease is a risk of living in the modern built environment. Since its discovery in 1976^12^, there have been numerous outbreaks of *Legionella pneumophilia* and related species^13^. The factors secreted by *L. pneumophilia* that enable it to live as a commensal bacterium in free-living amoebae, may contribute to its virulence in humans^80^. The situation is reminiscent of environmental *Mycobacteria* related to the *M. tuberculosis* complex which also live commensally in soil amoebae. If *Legionella* were to become readily transmissible from human to human, it could emerge as a twenty-first-century equivalent of tuberculosis.

D) The use of animal cells and tissues in humans—xenotransfusion and xenotransplantation—lays the recipient open to “perfect storm” conditions for zoonotic infections^81^. First, the physical barrier is bypassed with living animal cells or extracts inoculated or transplanted directly into the patient. Second, the human recipient is immunosuppressed. Third, to prevent hyper-acute rejection, the donor animal is genetically modified to knock out the “blood group” xenoantigen alpha-(1-3)-galactosyl-galactose, which may act as a natural barrier to enveloped viruses crossing from animals to humans^82^. To date, however, the gravest outbreak of disease resulting from xenotransfusion was less hi-tech: clients undergoing “rejuvenation” therapy at an alternative health clinic in Germany contracted fatal Q fever caused by *Coxiella burnetii*. It was present in the extracts of fetal lamb cells inoculated into the hapless recipients^83^.

## Selected historical epidemics and pandemics

Here, we present vignettes of the origins of selected epidemic incidents, juxtaposing some historical ones with modern equivalents. We discuss them in chronological order of appearance of the first recorded epidemic.

### The Plague of Athens

During the siege of Athens by the Spartans in 430 BCE, a grave epidemic appeared, as told by Thucydides in his *History of the Peloponnesian War*. To date, we do not know whether the disease was viral or bacterial; in fact, various scholars are still divided on the matter of its cause^84,85^. We are reasonably sure, however, that despite the label, it was not the disease we call the plague today. Two candidates consistent with the symptoms of the high fever, delirium, and dysentery described by Thucydides are typhus and typhoid fever. Typhus is caused by a *Rickettsia* with a reservoir in rats but is spread between humans by lice, whereas typhoid fever is caused by the bacterium *Salmonella typhii* transmitted by the fecal–oral route. Typhus became known as jail fever or war fever because of the unhygienic conditions in confinement or during conflict^86^. It decimated Napoleon’s *Grande Armée* in 1812 and was a major cause of death in Nazi concentration camps. Siege conditions also spurred other infectious diseases to progress into pandemics.

### Plague

The bubonic plague which famously caused the Black Death (1347–1352) is the pandemic that everyone, at least in the West, knows about from their schooldays^87^. The plague is one example of a disease of zoonotic origin, although even here we cannot be certain because the plague bacterium, *Yersinia pestis*, has an ancient association with humans. Recent analysis of ancient *Y. pestis* genomes, combined with archaeological and anthropological studies, has illuminated our understanding of repeated emergence of the plague since the Neolithic period^88^.

There have been three major, long-lasting plague pandemics in history, each with distinct *Y. pestis* genetic lineages^87,89^. Although all three pandemics are labelled rather specifically by geography or era, their true spatial and temporal scope are still matters of debate amongst historians of disease and medicine^89^. The first documented pandemic of plague known to be caused by *Y. pestis* arose in Western Eurasia in 541 CE and often is referred to as Justinian plague, after its Byzantine origins during the reign of the Emperor Justinian^90^. It erupted in the Egyptian port city of Pelusium and although the initial outbreak lasted only one year, the pandemic continued to spread and wreak havoc well beyond the Mediterranean world, as far north and west as Ireland in 664, until 750 CE^89,91^.

The Black Death is remembered as the archetypal pandemic that struck terror in the hearts of people in Europe and Mediterranean countries in which more than 35% people in affected areas died in 1348 alone. In fact, this first incident, which lasted from 1346 to 1353, marked the beginning of a series of outbreaks referred to as the Second Pandemic, which persisted as late as 1879^91^. With origins in the East—historians remain unsure whether it was in India, China, or the steppes of Russia—it travelled westward along the Central Asian Silk Road. It arrived in Western Europe in the autumn of 1347 on board ships docking at Messina from the Genoese trading post of Kaffa in Crimea. When the Genoese abandoned Kaffa, *Y. pestis* accompanied them and found a reservoir in black rats, which would reinfect humans in previously unaffected locales via their fleas.

The Third Pandemic is conventionally accepted to have started in Hong Kong in 1894^89^, although evidence points to an earlier origin in the Yunnan peninsula^91^. However, unlike the two earlier visitations, the spread of the third pandemic in the Old World appeared to be limited to coastal cities, except for China and India, where it exacted a massive toll, most notably in Bombay (now Mumbai) in 1896. Patterns of disease incidence appear to point to zoonotic transfers, in that the disease spread only when animal reservoirs survived and thrived. For instance, Wu Lien-Teh, who devised the modern face mask, surmised that the outbreak of the pneumonic disease in Manchuria in 1910–1911 was likely due to marmot hunting by fur trappers^92^.

The third plague pandemic arrived in San Francisco in 1900 in the poor Chinese precinct^93^. The Governor of California, Henry Gage, was concerned about the impact on his state’s economy and tried to ban press reports. He called it “the plague fake” in a letter to the US Secretary of State. But *Y. pestis* continued to fester among rats with sporadic human outbreaks. Following the 1906 earthquake, it colonized ground squirrels and chipmunks, in which it remains endemic today. Such animal reservoirs were likely responsible for the 1924 epidemic in the Mexican quarter of Los Angeles^1^. Meanwhile, in Buenos Aires, Argentina, where the disease had reached around the same time as San Francisco, large-scale fumigations with sulfur dioxide—which targeted both animal and insect reservoirs of the plague bacilli—proved effective in curbing the spread of plague^94^.

Although it has not caused any major pandemics since the early twentieth century, *Y. pestis* has not vanished. Examples of more recent plague outbreaks (for example, in Surat in India, the Azores, South America, and periodic eruptions in Madagascar^95^) support the idea that this disease is repeatedly zoonotic.

### Smallpox

Until its eradication in 1977, no other infectious disease had killed as high a proportion of the population in the past 500 years as smallpox (variola). Variola major has a case fatality of more than 20%, whereas variola minor has a case fatality of less than 10%^96^. Smallpox progenitors have reservoirs in burrowing rodents, although the proximate source of variola major probably transferred from Bactrian camels in East Asia, which in turn acquired it from gerbils during the early Christian era.

The outbreak of smallpox in 1520–1521 during the siege of Tenochtitlán (now Mexico City) was the pivotal factor by which Hernán Cortés and his band of Conquistadors vanquished the mighty Aztec empire, as recounted by Bernal Díaz in *The Conquest of New Spain*. It subsequently decimated indigenous populations in the Americas and Oceania^26^. In Europe and the US during the early eighteenth century, a particularly virulent variant of variola major led to epidemics which began to be controlled by adopting the Ottoman practice of variolation from the 1720s and by vaccination following Edward Jenner’s 1796 success with cowpox^41^.

### The English Sweats

“*A new kind of sickness came through the whole region, which was so sore, so painful, and sharp, that the like was never heard of to any man’s remembrance*”, a Tudor commentator wrote. From 1485 to 1551, the English sweating sickness appeared in five epidemics with a mortality of 30 to 50%^97^. In *Wolf Hall*, Hilary Mantel relates how suddenly Thomas Cromwell’s wife and children died in 1529. There has been much speculation about its cause. The symptoms and rapid onset are consistent with a hantavirus infection^98^, although there is no molecular evidence available for such an association. It resembles the 1993 Four Corners epidemic in the US, which was caused by a hantavirus “named” Sin Nombre virus^99^. The disappearance of the sweats after the mid-sixteenth century may have coincided with a population decline in a rodent reservoir.

### Syphilis

Syphilis, caused by *Treponema pallidum*, was first noted in Spain in 1493 and took off as a pandemic following the siege of Naples in 1495, in which Spanish mercenaries were employed by the besieging Bourbon commanders^100^. It rapidly spread across Europe and was exported by Portuguese navigators around the Cape of Good Hope and across the Indian Ocean to Asia, reaching Japan by 1510.

It seems most likely that syphilis was brought to Europe from Hispaniola by Christopher Columbus’s crew, but the debate remains unresolved despite extensive phylogenetic analyses of *Treponema* genomes^101^. Different subspecies are associated with yaws (*T.p. pertenue*), bejel (*P.t. endemicum*), and venereal and congenital syphilis (*T.p. pallidum*). Evidence of syphilis in pre-Columbian skeletons was unconvincing until recent study of specimens from Finland and Estonia which also detected a now-extinct *Treponema pallidum* strain in The Netherlands, basal to the yaws/syphilis split^102^. The authors argue, therefore, that progenitors were indeed present in pre-Columbian Europe. But that leads us to an intriguing thought that Norse explorers returning from the Canadian coast some 500 years earlier might also have imported syphilis to Nordic and Baltic areas!

Regardless of its earlier history, there is no doubt from contemporary chronicles that the features of syphilis that appeared in the 1490s represent a novel disease. Diaz de Isla wrote of his eyewitness account in Barcelona in 1493: “*It has pleased divine justice to send us unknown maladies, such as this serpentine sickness, which has never before been seen, or experienced or described in medical texts*”^100^.

Girolamo Fracastoro coined the term syphilis in his 1530 epic poem, *Syphilis sive de morbo gallico*. Here is a stanza from the 1686 translation by Nahum Tate:

*Say, Goddess, to what Cause shall we at last *
*Assign this Plague, unknown to Ages Past; *
*Perchance from Western Climes ‘twas wafted o’er, *
*When daring Spaniards sailed to their native shore; *
*From whence ‘tis grown so Epidemical, *
*Whole Cities’ Victims to its Fury fall; *
*Few ‘scape, for what Relief where vital Breath *
*The Gate of Life is made the Road of Death? *

In just four rhyming couplets, Fracastoro conveys the novelty of syphilis and speculates about its origin, rapid spread, and venereal transmission.

### Mosquito-borne viruses: yellow fever, West Nile encephalitis, and Zika

The prototype arthropod-borne flavivirus, yellow fever virus (YFV), is endemic in African monkeys and causes zoonotic infections. It took a century after Columbus for YFV to reach the New World, arriving in Barbados on board a slave ship, and it soon became sylvatic again, adopting New World monkeys as hosts in Central and South America. Although 80% of YFV infections are asymptomatic, severe disease made certain regions no-go areas and it is thought to have delayed the construction on the Panama Canal by 50 years. Human-to-human transmission of YFV via mosquitos engendered seasonal epidemics that routinely afflicted Americans during the eighteenth and nineteenth centuries. The worst documented encounter was in Philadelphia in 1793 when 50,000 people developed the disease with about 20% mortality^103^.

Since 1936, yellow fever has been controlled by a one-shot, live-attenuated vaccine, although its recent re-emergence in Brazil is a setback^104^. Other flaviviruses present twenty-first-century threats. Dengue virus is prevalent in cities where its vector, *Aedes aegypti*, thrives wherever small reservoirs of rainwater are present (for example, gutters and coconut shells). Moreover, dengue virus and the alphavirus, chikungunya, have adapted to a new vector, the tiger mosquito (*Aedes albopictus*), which used to be confined to Southeast Asia but has expanded its geographic range to Europe and the Americas^55^.

West Nile virus (WNV) and Zika virus are flaviruses that were first isolated in East Africa in the 1940s and have recently colonized the Americas. In 1999, a neurovirulent strain of WNV appeared in New York City, causing encephalitis in people and crows. It rapidly spread as far as the West Coast^105^ and is now endemic coast to coast and from Canada to Venezuela. WNV is also spreading in Europe, where there were 285 cases and 31 deaths in 2020. Most cases are primary zoonoses from birds vectored by *Culex* mosquitos. The genome sequence of the 1999 New York strain is almost identical to one in Tel Aviv, indicating a Middle East origin^106^, but whether WNV flew across the Atlantic in a bird, a human, or a stowaway mosquito remains unknown. Regarding the avian reservoir^107^, the more urban the environment, the more prevalent the infection, probably because the species of *Culex* mosquito vectors thrive in the human habitat^108^. WNV is now the leading cause of mosquito-borne disease in the US. Its arrival in the New World at the end of the twentieth century resonates with yellow fever 350 years earlier.

Zika virus is related to the four dengue virus subtypes and for 60 years had not been associated with epidemic disease^109^. In 2014, Zika virus re-emerged in Micronesia. It crossed the Pacific Ocean and spread pandemically in Latin America, where it was linked to microencephaly in babies infected *in utero*^109^. Unlike WNV but as with dengue, Zika is spread by *Aedes* mosquitos directly between humans rather than relying on an animal reservoir^110^. Its pandemic spread after it reached Brazil was explosive^111^.

### Cholera

Cholera is an example of an infectious disease that cannot be ascribed to zoonotic origins either now or in history. It is caused by *Vibrio cholerae* bacterium, indigenous in the Ganges–Brahmaputra delta in Southeast Asia (Colwell^4^), where it has been endemic for centuries if not millennia and where the bacillus is often found in commensal association with algae^112^. Cholera did not emerge in pandemic proportions until 1817^113^. This sudden and massive spread is generally attributed to the dramatic rise in the scale and rapidity of human movements—usually troops—in the early nineteenth century during the introduction of faster mass transport systems, such as steamships and railways. There have been seven cholera pandemics since 1817, although there is no consensus about the exact dates of and intervals between the different episodes^113^. It was during the third pandemic (1852–1859) that British physician John Snow mapped the source of the disease to the notorious Broad Street pump^114^ and later in south London^115^, which supported the hypothesis that cholera was a water-borne disease and not caused by miasmas^116^.

Robert Koch isolated and identified the causative bacterium during the fifth pandemic (1881–1896)^117^. It is generally accepted that this strain of *V. cholerae* was responsible for the first six pandemics, whereas the seventh pandemic, which originated in 1961 in Indonesia and is estimated even now to affect 3 to 5 million people annually^118^, was caused by the *El Tor* strain^4^. Genetic fingerprint analysis has confirmed that different *V. cholerae* variants are endemic in different geographic regions, forming pockets of potentially new pandemic threats^119^. Mobile genetic elements such as bacteriophages and plasmids encoding cholera toxin genes influence the colonization and severity of the disease^120^.

### Anthrax

Whether anthrax should be regarded as zoonotic or coming from free-living microbes is a moot point. Human cases are most frequently acquired from the corpses or products of livestock, but the reservoir for anthrax is the soil where spores of *Bacillus anthracis* can survive for many decades. Contact with contaminated animal tissue leads to cutaneous anthrax, and inhalation of spores leads to the more fatal pulmonary disease. Historically, inhalation of anthrax spores was called woolsorters’ disease as it was an occupational hazard^121^.

### Polio: the most feared epidemic of the mid-twentieth century

**Figure 5.**
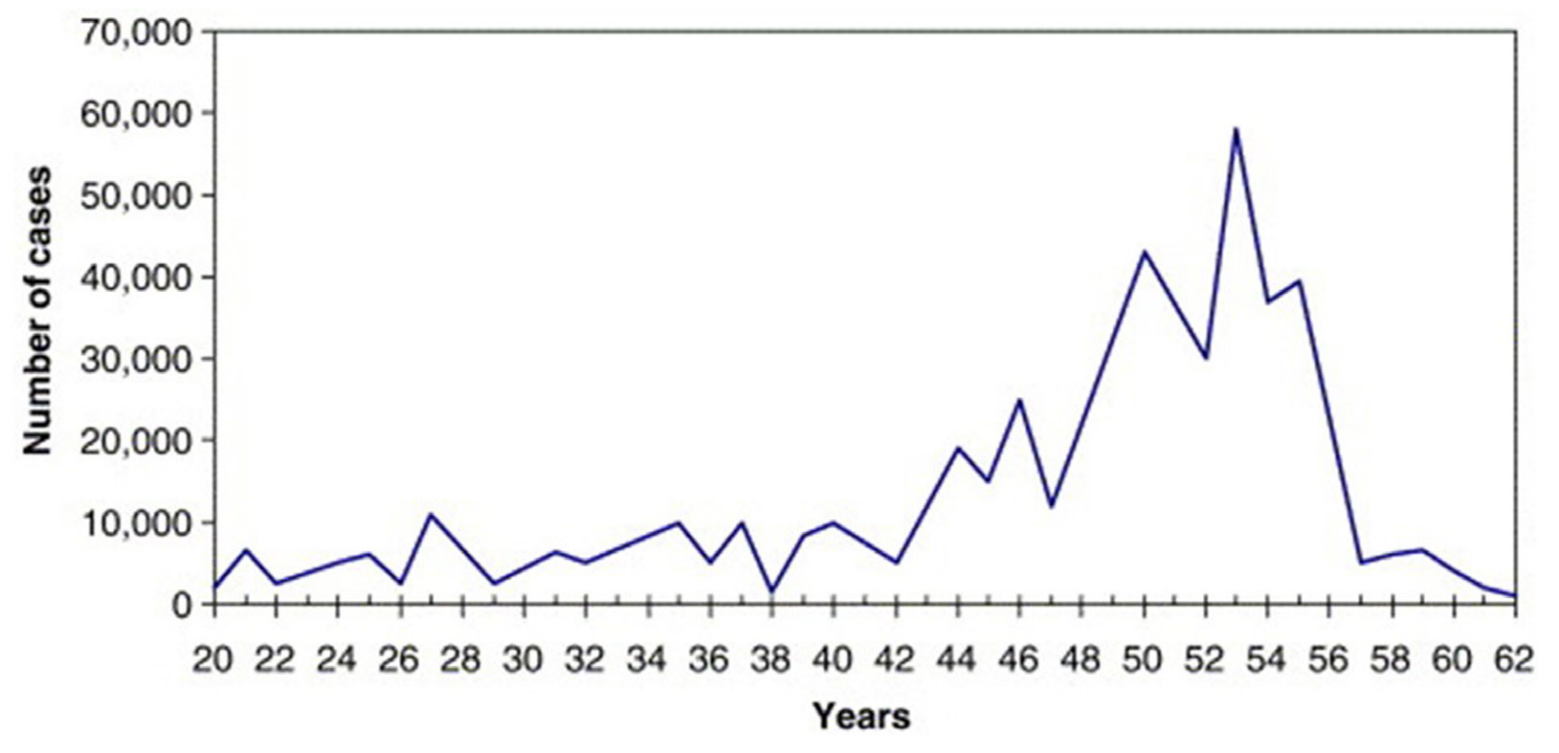
The polio epidemic. Cases of symptomatic (mainly paralytic) poliomyelitis in the US from 1920 to 1962. The steep fall from 1955 shows the protective power of the Salk vaccine introduced that year. Figure reprinted from Journal of Theoretical Biology, 237, Bunimovich-Mendrazitsky S and Stone L, Modeling polio as a disease of development, 302–315, 2005^122^ with permission from Elsevier.

Poliomyelitis is a debilitating and sometimes fatal paralytic disease. It is caused by three related enteroviruses called poliovirus types 1, 2, and 3. They are transmitted by the fecal–oral route and have been endemic in humans since ancient times. Polio emerged in epidemic form during the twentieth century^122^ mainly among middle-class children and adults. During the summer of 1952 in the US, 57,000 people were affected, 21,000 were paralyzed, and 3,145 died. Swimming pools and movie theaters were closed and parents isolated their children at home^123^. On April 12, 1955, the success of Jonas Salk’s inactivated poliovirus vaccine was announced, curtailing the annual epidemics (Figure 5), followed by Albert Sabin’s live-attenuated vaccine in 1960.

Paradoxically, the risk of polio as a disease mushroomed as sanitation in industrial societies improved since the odds of acquiring poliovirus early in infancy diminished for families living in hygienic conditions. Newborn babies seldom develop paralytic polio probably because of the presence of low levels of protective maternal antibodies which even when they do not stop infection can prevent spillover from the gut to the central nervous system. The infants then develop their own immunity to the infection. Better sanitation created a sizable middle-class population of susceptible children and adults for epidemics to emerge from a reservoir in lower socioeconomic groups.

Polioviruses are now on the brink of global eradication. But revertants to virulence emerge from live-attenuated vaccines^124^, particularly if polio type 2 is still present in the vaccine. This cautionary tale illustrates how an age-old, universal endemic infection with low disease incidence exploded into annual epidemics as herd immunity was lost. It also shows the power of vaccines to reinstate herd immunity and eventually eliminate the disease.

### Influenza

Influenza is a group of viruses that repeatedly spills over from wildfowl reservoirs and often appears to reach humans via an intermediate host in livestock (Figure 2). The virus has a high mutation rate leading to antigenic variation (genetic drift) and can also re-assort its eight genome fragments to generate new, recombinant strains (genetic shift) which may combine an expanded host range with virulence^125^. Whereas influenza A viruses give rise to mild, enteric infections in wildfowl, in poultry and mammals they adapt to a more virulent mode of respiratory transmission and in some cases have re-seeded wildfowl reservoirs with more highly pathogenic variants. One anomaly is the highly pathogenic H5N1 strain that emerged in 1997 and devastated domestic chicken flocks, was also lethal to the few people who became infected. Fortunately, however, it has not yet become readily transmissible between humans. H5N1 influenza is an unusual and worrying example of a virus that emerged among domestic fowl and then spread to wildfowl.

The biggest impact of influenza in modern history was the notorious 1918–1919 influenza pandemic known as Spanish flu, which resulted in more than 50 million deaths^126^. Unlike in COVID-19 and seasonal flu epidemics, however, elderly people were relatively protected, perhaps because they retained a degree of immunity from a previous infection by a related flu strain.

### AIDS

AIDS was first documented in 1981 in the US but came from central Africa. The mortality due to HIV infection since then (~40 million deaths) makes AIDS the most severe pandemic since Spanish flu in 1918–1919. AIDS mainly strikes young adults, so the social and economic consequences of its mortality are immense. In addition, the stigma frequently attached to people with HIV infection, and to the risk groups prone to infection, has greatly exacerbated the problems of managing HIV/AIDS. We have discussed some of these problems elsewhere^127^. Here, we focus on the origins of human AIDS. Four lineages of HIV-1 (groups M, N, O, and P) arose as separate zoonotic transfers from African apes^128^. The pandemic lineage, group M, has a genome sequence closely related to SIVcpz endemic in chimpanzees in Southeast Cameroon. Pepin^42^ cogently argues that colonial exploitation of subject peoples promoted HIV-1’s journey down river to Leopoldville (Kinshasa). There, it festered for 50 years^129^ before expanding into a pandemic in East Africa at around the same time as it appeared in the US (probably via Haiti). It then spread explosively in Southern Africa in the 1990s. Like hepatitis C virus, HIV-1 gained a wide dissemination through blood transfusions and the pooling of contaminated blood products such as clotting factor VIII before HIV screening of donations was introduced. Despite our failure to develop an efficacious vaccine, anti-retroviral drugs have greatly helped not only to reduce mortality but also to turn the tide on transmission rates.

**Figure 6.**
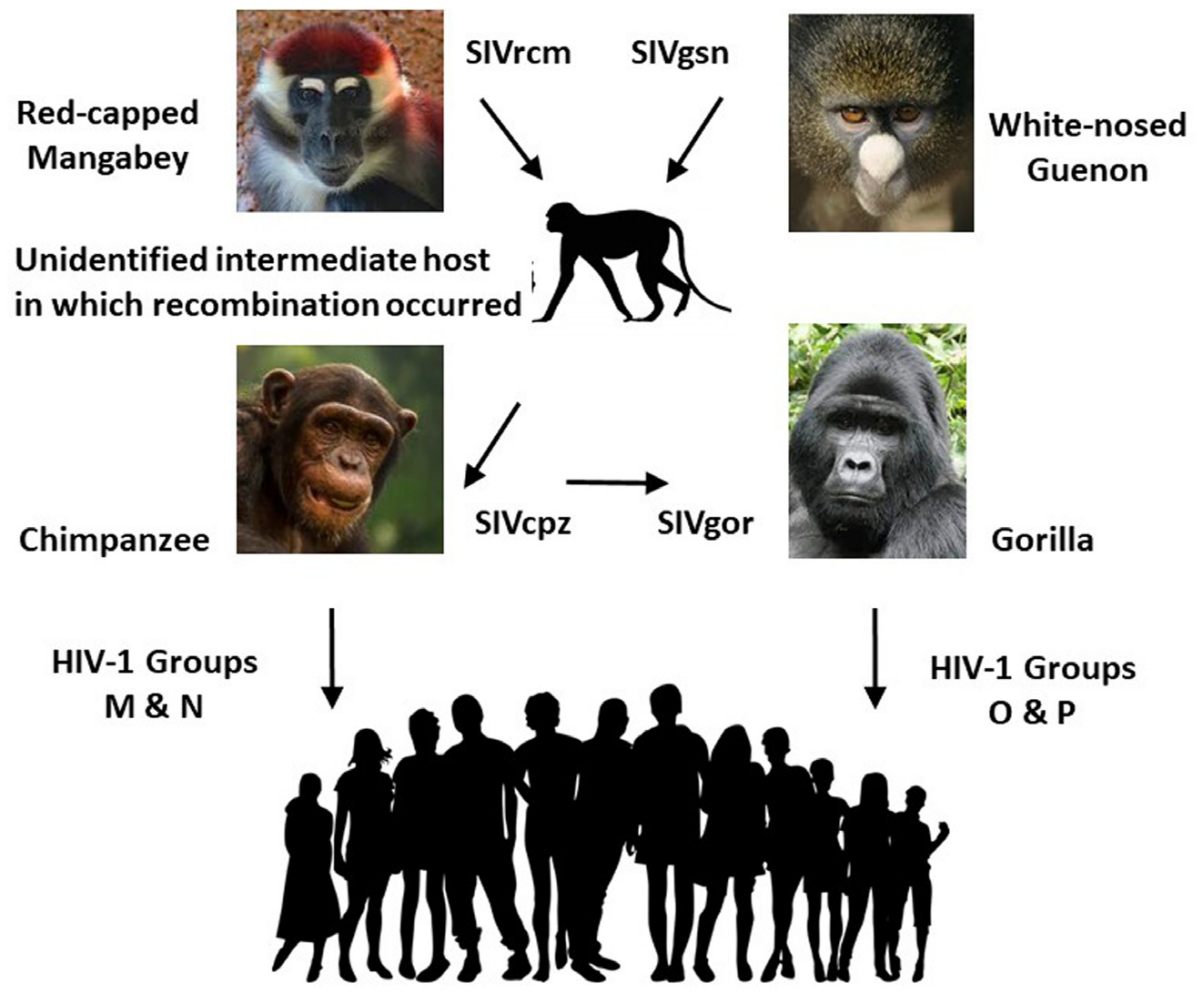
The complex evolution and origins of HIV-1. Many species of African primate harbor simian immunodeficiency viruses (SIVs) which are not present in Asian or New World primates. The proximate reservoirs of HIV-1 were the SIVs of chimpanzees (SIVcpz), giving rise to HIV-1 groups M and N, and gorillas (SIVgor), giving rise to HIV-1 groups O and P^130^. These in turn probably came from an unidentified monkey species in which genetic recombination occurred between SIV strains of the red-capped mangabey and the white-nosed guenon^131^. Two subspecies of chimpanzee are reservoirs of SIVcpz, the Central African *Pan troglodytes troglodytes* and the East African *P. t. schweinfurthii*, but no human transmissions are known from the latter, whose SIV is inhibited by human cellular restriction factors. The infection of lowland gorillas is more likely to have come from *P. t. troglodytes* than directly from the intermediate host because it is more closely related to that of *P. t. troglodytes* than that of *P. t. schweinfurthii*. Only HIV-1 group M generated a pandemic, although HIV-1 group O infected up to 10,000 people before regressing. (Copyright-free images from Pixabay)

In contrast to the cross-species transmission of HIV-1 group M from chimpanzees, group O originated in lowland gorillas^130^. Prior to reaching the great apes, the evolution of HIV-1 precursors (simian immunodeficiency virus, or SIV) through different primate hosts was complex^131^ (Figure 6). Of the four zoonotic events from apes that became HIV-1 (and 10 SIV transfers from sooty mangabey monkeys to become HIV-2), only HIV-1 group M moved from *Pan* to pandemic. From the virus’s point of view, it was a lucky break; viruses that adhere too strictly to their original host are themselves doomed if that host becomes extinct. This one has jumped from an endangered subspecies of chimpanzee of about 125,000 individuals^130,132^ to a human population of 7.8 billion—and has already infected 75 million of them.

### COVID-19 and other coronaviruses

With an estimated 50 million cases and 4.5 million deaths worldwide^133^ since it emerged in December 2019, COVID-19 is the largest pandemic since AIDS. SARS-CoV-2, the agent of COVID-19, represents one of nine coronaviruses known to jump to humans^134^; the seven endemic and epidemic strains are shown in Table 2. It was identified in January 2020^135^ as a member of the sarbecovirus subfamily of coronaviruses. Its first appearance in humans in the Hubei province of China is estimated to have occurred in October or November of 2019^31^. Other twenty-first-century outbreaks of highly pathogenic coronaviruses are SARS, the prototype sarbecovirus, and MERS. A key difference between SARS-CoV-1 and SARS-CoV-2 is that the former is not infectious prior to severe symptoms, which helped to contain the epidemic. One of the common cold viruses, OC23, of probable cattle origin, may have caused a severe epidemic with COVID-19-like symptoms in 1890^136^ and appears to have become attenuated. Perhaps COVID-19 will take a similar path.

**Table 2.** Seven human coronaviruses that originated from four distinct lineages.

Virus	Coronavirus type	Proximate source	Reservoir species	Date of zoonosis^a^	Cellular receptor
Common cold viruses:					
229E	Alpha	Bovine	Bat	~1500	APN
NL63	Alpha	?	Bat	~1820	ACE2
OC43	Beta lineage A	Bovine	Rodent	1889	SA
HKU1	Beta lineage A	None?	Rodent	~1950	SA
Severe respiratory syndrome viruses:					
MERS	Beta lineage C	Dromedary	Bat^b^	2012	DPP4
SARS	Beta lineage B	Palm civet cat	Bat^c^	2002	ACE2
SARS-CoV-2	Beta lineage B	None?	Bat^d^	2019	ACE2

^a^Precise dates are based on outbreaks, and approximate dates are based on molecular clock estimates of most recent common ancestor between human and reservoir viruses. ^b^Pipistrelle subfamily. ^c^Chinese horseshoe bat, *Rhinolophus sinicus*. ^d^Unknown *Rhinolophus* species. ACE2, angiotensin-converting enzyme type 2; APN, aminopeptidase N; CoV, coronavirus; DPP4, dipeptidylpeptidase; MERS, Middle East respiratory syndrome; SA, sialic acid, SARS, severe acute respiratory syndrome.

The origin of SARS-CoV-2 has not been definitively identified but as already discussed is probably a species of horseshoe bat (*Rhinolophus* spp)^137^. The precise route taken prior to its appearance in Wuhan remains to be determined, and there is some debate over natural infection of a human or the less likely release from the Wuhan Virology Institute^134^. Perhaps in reaction to conspiracy theories promulgated in the West, Chinese authorities have been too vehement in denying even the possibility of inadvertent escape from the Institute’s coronavirus laboratory, for there is a precedent with the escape of SARS-CoV-1 in Beijing^138^. We conjecture another possibility that would explain the coincidence of the outbreak of COVID-19 in the very city where multiple SARS-related viruses were under study, namely that a field researcher might have acquired the infection directly from the bat samples being collected.

Because SARS-CoV-2 infected many people associated with the wet market selling live animals, it might have reached humans via an intermediate host in the market, similar to the masked palm civet cat (*Paguma larvata*) (as much an inhabitant of rice fields than forests) in the SARS outbreak in Guangdong province in 2003^139^. On the other hand, it has become apparent that wet markets and abattoirs provide an excellent humid atmosphere for the human-to-human spread of SARS-CoV-2 via contaminated aerosols^140,141^ and therefore transmission in the Wuhan market may represent a subsequent amplification step in its emergence.

In experimental studies^142^, SARS-CoV-2 can infect several domestic animal species, and the COVID-19 pandemic has provided opportunities for zoo-anthroponosis of SARS-CoV-2 from humans to animals. A variant has spread widely among farmed mink in The Netherlands and back to humans^21^ and it similarly led to the cull of 17 million mink in Denmark. Thus, where humans have contact with susceptible animals, those species may be at risk of acquiring COVID-19. Zoo-anthroponosis is a particular threat to endangered species; the human coronavirus OC23, as well as other human respiratory viruses^143^, is on record of jumping to wild chimpanzees^144^ due to eco-tourism.

Although the typical R_0_ value or transmission rate of SARS-CoV-2 is not as high as that of, say, measles, it is proving difficult to suppress in societies where people hold their individual liberty to be more important than community health or when governments issue mixed messages. The recent emergence of even more transmissible variants of SARS-CoV-2 shows the ongoing adaptation of the virus to its new human host^145^. Such variants have independently emerged in the UK, South Africa, Brazil, and India and have rapidly replaced the initial human strains. Hopefully, existing and modified vaccines will protect against these variants; otherwise, it will not augur well for control of the pandemic.

## Concluding remarks

In this article, we have emphasized the great variety of origins of epidemics geographically and ecologically, including risks of advanced technologies. Contrary to popular belief, many of the species providing zoonoses are not spillover infections from the distant habitats but are our “companion” animals living in the human biosphere, such as rats and bats.

Once an epidemic takes off and expands to become pandemic, however, there are features in common with historic epidemics in the struggle to achieve containment. We should be humbled by the fact that we still rely on traditional means to attempt to contain the COVID-19. We use quarantine methods (self-isolation) dating from Venice’s approach to the Black Death 670 years ago, social distancing reminiscent of those imposed on lepers, and 50-year-old methods of contact tracing for smallpox. In 1847, Hungarian physician Ignaz Semmelweis, having observed that childbed fever in doctors’ wards had three times the mortality of midwives’ wards, implored his unheeding colleagues to “wash their hands” as they made their way from the morgue to the delivery room. Face masks, first devised by Wu Lien-Teh to protect people from pneumonic plague in Manchuria in 1910, were widely used during the 1918–1919 influenza pandemic and resemble those worn today^90^.

Nonetheless, we can marvel at the acceleration of modern research methods to aid practical developments in disease control: The entire genome sequence of SARS-CoV-2 was accomplished by Chinese scientists within a week of the virus’s identification. The immediate release of the sequence into the public domain led to international tracking and accurate diagnostic tests of infection within another week; it was also the key information to kick-start the development of all the current COVID-19 vaccines. The new normal through the experience of this pandemic may enable us to shorten drug and vaccine development and regulatory approval, not only to turn the tide against COVID-19 but also to set a model for other diseases – so long as the even more rapid dispersal methods of misinformation, false rumors, and conspiracy theories against vaccines don’t stymie our efforts.

We should also reflect how drastically wrong the experts proved to be on pandemic preparedness. In October 2019, the highly respected Global Health Security Index^146^ compiled by the Bloomberg School of Public Health at Johns Hopkins University placed the US and the UK at the top of a list of 195 countries considered the best prepared to handle “*Rapid response to and mitigation of the spread of an epidemic*” on account of their surveillance expertise. In practice, however, by doing too little too late, these two countries have ranked among the worst for high incidence and fatality rates of COVID-19. With over 700,000 deaths by the beginning of October 2021, the US has the highest recorded per-capita COVID-19 mortality. Other nations, despite having more contact with mainland China where the disease first appeared, showed that the pandemic can be mitigated. For instance, Taiwan, an island democracy of 24 million people, reported 842 COVID-19 deaths by the end of September 2021; in contrast, the UK, an island democracy of 68 million people, recorded 136,000 deaths by that date^133^. The Taiwanese government took prompt and decisive action to curtail flights from mainland China as soon as news of the Wuhan outbreak was announced, and their citizens were compliant on quarantine restraints.

We have briefly reviewed the nature of epidemics from the Plague of Athens in 430 BCE onwards and how globalization via the Silk Road across Asia and worldwide navigation created baleful pandemics such as the Black Death and smallpox. New epidemics will, of course, continue to emerge and their origins may surprise us. Perhaps what is most notable in the twenty-first century is the syndemic aspect of the knock-on effects of novel infections in our interconnected yet fragile society. Thus, COVID-19 revealed that even with sophisticated surveillance systems, we remained as ill prepared to prevent the disruption to our lives and livelihoods as our forebears who faced pandemics with much higher mortality rates.
